# The Use of Photoplethysmography in the Assessment of Mental Health: Scoping Review

**DOI:** 10.2196/40163

**Published:** 2023-05-29

**Authors:** Lynnette Nathalie Lyzwinski, Mohamed Elgendi, Carlo Menon

**Affiliations:** 1 Faculty of Health Sciences Simon Fraser University Burnaby, BC Canada; 2 Menrva Research Group Schools of Mechatronic Systems Engineering and Engineering Science Simon Fraser University Vancouver, BC Canada; 3 Biomedical and Mobile Health Technology Lab Department of Health Sciences and Technology, ETH Zurich Zurich Switzerland

**Keywords:** photoplethysmography, PPG, mental health, depression, anxiety, suicide, mobile phone

## Abstract

**Background:**

With the rise in mental health problems globally, mobile health provides opportunities for timely medical care and accessibility. One emerging area of mobile health involves the use of photoplethysmography (PPG) to assess and monitor mental health.

**Objective:**

In recent years, there has been an increase in the use of PPG-based technology for mental health. Therefore, we conducted a review to understand how PPG has been evaluated to assess a range of mental health and psychological problems, including stress, depression, and anxiety.

**Methods:**

A scoping review was performed using PubMed and Google Scholar databases.

**Results:**

A total of 24 papers met the inclusion criteria and were included in this review. We identified studies that assessed mental health via PPG using finger- and face-based methods as well as smartphone-based methods. There was variation in study quality. PPG holds promise as a potential complementary technology for detecting changes in mental health, including depression and anxiety. However, rigorous validation is needed in diverse clinical populations to advance PPG technology in tackling mental health problems.

**Conclusions:**

PPG holds promise for assessing mental health problems; however, more research is required before it can be widely recommended for clinical use.

## Introduction

### Background

Photoplethysmography (PPG) is an emerging technology that uses light to measure microvascular blood changes to assess various cardiovascular parameters that are useful in clinical settings [1,2], including blood pressure [3,4] and heart rate (HR) [5]. This technology can quickly and easily assess vasoconstriction and dilation in the skin, autonomic function changes, and vascular functions [6]. PPG assesses these physiological changes through a clip placed on a body part, such as the finger or ear [7,8], and smartphone apps [5,9].

In recent years, PPG technology has been extended to include the assessment of mental health changes in patients [10], which is of great pertinence to patients who experience challenges in accessing mental health care. Many individuals, particularly those living in remote areas, struggle to obtain equitable access to mental health services [11]. Mental health disparities and the need for mental health interventions in remote settings were especially pronounced during the COVID-19 pandemic [12]. In addition, an increase in mental health problems [13] and the risk of suicide [14] has been of concern during the pandemic era, stemming from the lack of social support (interpersonal theory of suicide) [15]. It is well-known that mental health problems are linked to an increased risk of cardiovascular disease through direct effects on the myocardium, including increased HR and blood pressure [16-19]. PPG may help provide a rapid mental health assessment for patients by assessing changes in HR variability (HRV), and a subsequent medical referral to a specialist can be made [3]. Rural and remote patients might possibly also feel greater support if PPG is provided alongside remote telemental health consultations [20].

As PPG is an emerging technology and its application in assessing mental health problems has only recently been used, its overall effectiveness, accuracy, and challenges are not entirely clear. There is a need to determine whether PPG is an effective technological medium for the screening and prediagnosis of mental health problems as a complement to the currently used methods, including clinical evaluations based on self-reported symptoms [21]. In other words, PPG may be used by a wide range of mental health practitioners as a useful adjunct tool. For example, mental health professionals may use it as a prescreener before making referrals. In addition, psychiatrists, psychologists, and other allied health professionals [22] may use it to monitor patients’ symptoms in a remote setting.

There is also a need to evaluate how PPG-based technology can be integrated into current standard methods for diagnosing patients with mental health problems. Currently, standard guidelines require practitioners to assess patients using the fifth edition of the Diagnostic and Statistical Manual of Mental Disorders (DSM-5) [23]. Diagnosis is usually face-to-face but may be virtual through telehealth for remote patients [20]. A crucial research question is whether PPG is a valid and reliable method for assessing mental health and psychological problems. In other words, how much in-depth information can PPG blood flow changes offer compared with traditional face-to-face assessments by mental health professionals for assessing mental health? The other question is whether PPG can be used alone (without requiring questionnaires) for screening. Furthermore, there is also a need to determine what types of studies have been conducted on PPG and mental health, and whether there is any evidence supporting the use of this technology for managing mental health problems.

### Objectives

The objectives of this review were as follows:

1. To identify the types of PPG studies conducted on mental health. Have any smartphone apps (mobile health approaches) been developed for mental health using PPG?
2. To determine the effectiveness of PPG for assessing, screening, or monitoring common mental health problems [24] (with an emphasis on depression and anxiety). Symptoms relevant to mental health, such as stress, are also of interest.
3. To assess the validity and reliability of the PPG-based mental-health methods
4. To identify challenges associated with this new technology and make recommendations for future research

Our main outcome of interest was efficacy and accuracy (validity and reliability) in screening for depression, anxiety, or psychological distress in individuals. In other words, how accurately can PPG assess the mental health end point? The secondary outcomes of interest were any identified barriers or challenges with existing PPG-based technologies proposed for mental health problems.

## Methods

### Overview

PubMed and Google Scholar were searched for articles published in January 2021. The search terms included word variations for PPG and mental health including depression, anxiety, or stress. The search strings were combined into one large search string that was the most relevant to PPG and mental health. Keywords included Medical Subject Headings (MeSH) terms, free text, word variations, and truncation. We discussed the search strategy, including keyword variations, with a medical librarian during the search planning phase. In addition, manual searches of articles meeting the inclusion criteria were performed to identify additional articles. It should be noted that we reran the search, with the term “photoplethysmogram” again in December 2022, to determine if any studies were missed in PubMed. This generated 33 additional results that were irrelevant.

### Inclusion and Exclusion Criteria

The literature search was limited to articles published over the past decade. The search strategy is defined as follows: “Photoplethysmography”[Mesh] OR Photoplethysmography[tiab] OR PPG [ti] AND “Depression”[Mesh] OR depression[tiab] OR depress*[tiab] OR “anxiety”[mesh] OR “anxiety”[tiab] OR “Mental Health”[Mesh] OR “Stress, Psychological”[Mesh] OR stress[tiab] OR “Quality of Life”[Mesh] OR quality of life[tiab] OR QoL[tiab] OR well-being[tiab].

All intervention studies were included if they evaluated mental health conditions or symptoms in relation to the PPG. Studies must have evaluated clinical mental health problems, such as depression, anxiety, or schizophrenia, without other comorbidities. Studies that evaluated psychological stress were included only if they were in a population with a history of a clinical mental health problem, especially anxiety. High psychological stress has been linked to anxiety and depression [25,26]; hence, evaluating it in patients with clinical anxiety is relevant for monitoring its progression. Stress is also a risk factor for mental health problems and can be managed through psychological interventions [27,28]. In addition, studies evaluating other symptoms in patients with mental health problems that came up in the search were included if they involved intrusive thoughts or suicidal thoughts and suicidal ideation. There has also been a consideration for including suicidal behavior as a separate mental health disorder in the DSM-5. General stress studies were included only if they evaluated at least one mental health disorder in clinical populations, particularly anxiety. Furthermore, the studies must have used a screening tool for diagnosing anxiety, such as the General Anxiety Scale [29], when comparing it with PPG. General studies on psychological stress in nonclinical populations and physiological stress were excluded. Studies involving participants diagnosed and treated by clinicians in a face-to-face setting (nontechnological) or over telehealth without the assistance of artificial intelligence for advancing technology such as PPG (this allows a comparison of the accuracy of diagnosis) were excluded. Studies that did not use PPG technology and those that used PPG technology for purposes other than mental health assessment or preassessment, complementary screening or press-screening, and monitoring were also excluded. Finally, the studies had to have been published in the English language.

### Screening

The citations of the included papers were imported into EndNote (version X7.1; Clarivate). Relevant titles were screened, followed by abstract screening of titles that met the inclusion criteria. Two independent reviewers, LNL and ME, screened the abstracts to ensure that the articles fulfilled the inclusion criteria. Once there was an agreement, full texts were retrieved from the articles that met the inclusion criteria. Where there was any disagreement between the reviewers, the articles in question were discussed until agreement was reached.

### Data Extraction and Synthesis

Data on the general study characteristics and participant demographics were extracted and summarized in a tabular format. These data included the authors, years of publication, study design, country, participants (age and sex [male or female]), mental health history, anatomical site of PPG measurement, and mental health measures (anxiety, stress, depression, relaxation, and happiness). It also included outcomes, such as the effectiveness of PPG for diagnosing depression, anxiety, or stress. Other outcomes included data on the validity and reliability of PPG (eg, Bland-Altman plots comparing PPG with standard methods of diagnosis), as well as qualitative data on barriers associated with PPG technology. This review followed the PRISMA-P (Preferred Reporting Items for Systematic Review and Meta-Analysis Protocols) guidelines [30].

## Results

### Search Results

**Figure 1:**
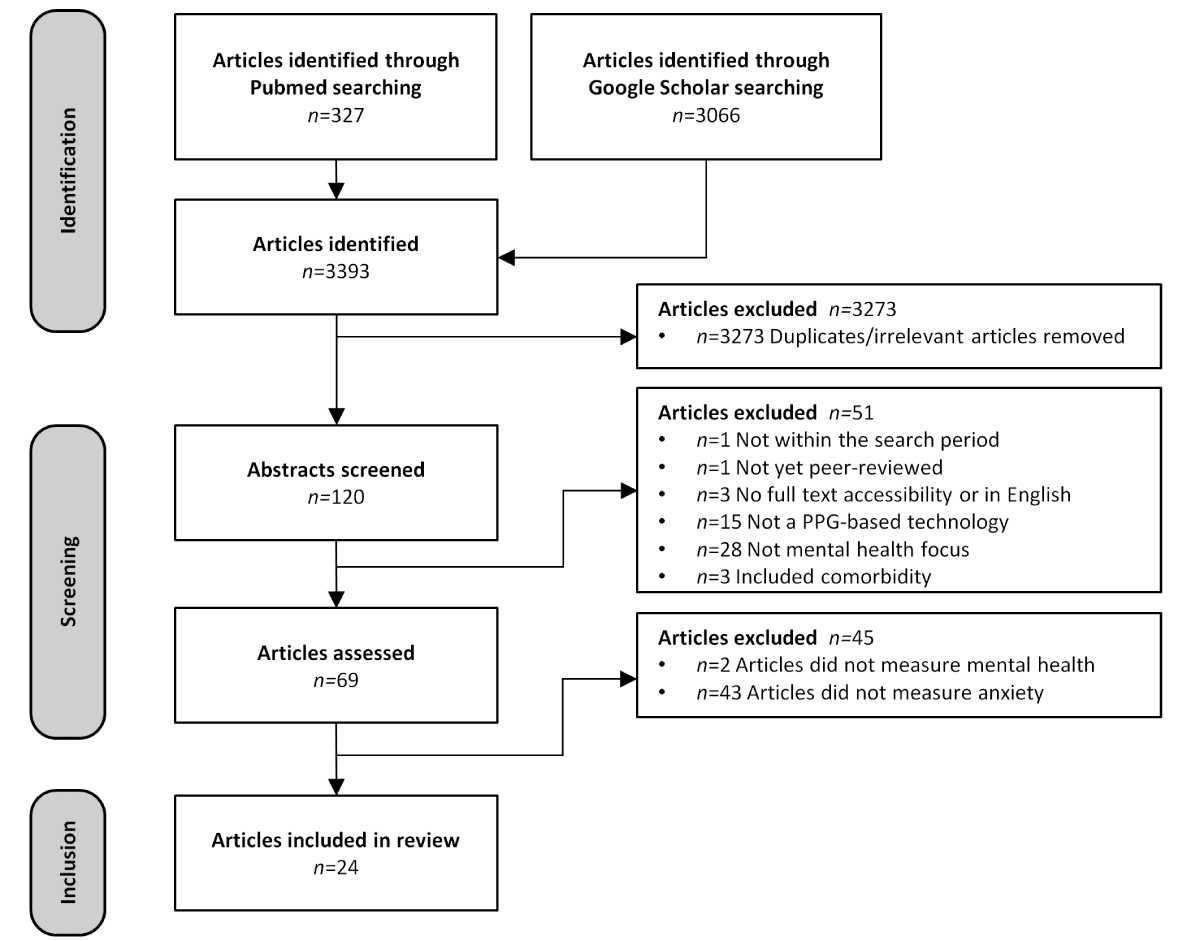
Summary of articles included and excluded in this study. PPG: photoplethysmography.

The details of the search process are shown in Figure 1. We identified 3393 records. After title screening and review of 114 abstracts against the inclusion and exclusion criteria, followed by full-text retrieval, 24 studies were included in the review [31-54]. It should be noted that the 2 sets of studies were sister papers [32,37,38,51,52]. The sister papers are those authored by the same author who published multiple articles using the same data set. However, each article is treated independently in this study.

The main characteristics of the included articles, including mental health conditions, diagnostic methods if provided, and population (including the control group, if provided), are described in Table 1.

The studies were conducted in Japan [33,40,51,54], China [44,45], South Korea [52], Abu Dhabi [38], Taiwan [43], Spain [42], Turkey [31], Sweden [39], Canada [35], Germany [32,53], and the United States [41,48]. Most of these were proof-of-concept studies with a small number of participants. Three studies had a control group with sample sizes ranging from 61 to 308 [37,42,52]. The age range was broad across the studies, ranging from 16 to 85 years. The studies were relatively balanced in terms of sex (male or female), although not all reported this information. One study was conducted only on female participants [47].

Most studies used finger-based PPG methods to evaluate mental health outcomes and psychological stress [31-33,38,39,43,46,49,51]. Some researchers have used this arm for PPG measurement [10]. A few have used smartphone-based technology, which includes a PPG finger sensor and video camera [35,44] or face-based webcam technology [54]. This implies that mobile methods, including smartphones, for PPG are scarce and are emerging as novel methods for assessing mental health.

**Table 1:** Study characteristics.

Year	Study	Sample size, N	Sex ratio (% F^a^)	Age range (years)	Mental health measures	Gold standard	Comorbidity or confounding factors	Evaluation metric (mental health vs control)
2022	de Vries et al [34]	9	22.3	26-52	Depression and anxiety	4DSQ^b^	To account for confounders, control variables for TST^c^, MVPA^d^, and alcohol use were included	Association between HRV^e^ fluctuations and stress increase
2021	Sigrist et al [53]	27	100	13-17	Borderline personality disorder	SCID-II^f^, severity of psychopathology (CGI-S^g^), and GAF^h^	Controlled for the potential confounders of age, BMI, menstrual cycle, cardioactive medication, oral contraceptives, smoking behavior, alcohol consumption and drug consumption, physical activity level, and illness	HR^i^ significantly correlated with gold standard with *P*<.05
2021	Long et al [45]	36	53	22-43	Depression	PHQ-9^j^	N/R^k^	Developed a classifier to differentiate between depressed and health subjects with accuracy=85.3%
2021	Harvie et al [35]	104	35	17-50	Stress	TSST^l^	N/R	Significant acceleration in HR and heightened self-reported stress and anxiety in the TSST condition relative to a placebo version of the TSST with *P*<.05
2021	Unursaikhan et al [54]	53	42	18-60	MDD^m^	SDS^n^	N/R	HRV and SDS correlated significantly (*P*<.05) and can detect MDD with sensitivity=73% and specificity=85%
2021	Kontaxis et al [42]	80	60	32-59	MDD	HDRS^o^ and BDI^p^	N/R	HR and amplitude difference between systolic and diastolic peaks are significantly different in healthy and MDD subjects
2021	Kimmel et al [39]	126	100	22-43	Panic disorder, phobia, trait anxiety, and traumatic events	EPDS^q^ and the STAI^r^	Comorbidities were reported	LF^s^/HF^t^ ratio significantly correlated with anxiety with *P*<.001
2021	Moshe et al [48]	55	54.5	41-54	Depression and anxiety	DASS-21^u^	N/R	HRV and anxiety are significantly correlated with *P*<.05
2020	Perpetuini [50]	102	47	20-70	Anxiety	STAI-Y^v^	N/R	HRV and STAI-Y score are correlated with *r*=0.81
2020	Liu et al [44]	93	54	21-28	Depression	SWLS^w^	N/R	HRV significantly differentiates between high and low SWLS scores with *P*<.05
2020	Ismail et al [36]	34	61.8	20-27	SAD^x^	DSM-5^y^ and LSAS^z^	N/R	HR can detect SAD with accuracy=88.9%
2019	Mistuhashi et al [47]	7	28.5	22-24	Stress and trait anxiety	N/R	N/R	HR detects stress with accuracy=80%
2018	Ngamprmuan et al [49]	40	65	20-43	MDD	Wisconsin Card Sorting Test	N/R	HRV significantly differentiates between MDD and control subjects with *P*<.001
2018	Koening et al [41]	90	67.8	14-19	MDD	Clinicians’ ratings of depression severity and BDI-II	Comorbidities were reported	HRV significantly differentiates between depression and control subjects with *P*<.05
2018	Dagdanpurev et al [33]	20	50	23-60	MDD	N/R	N/R	Developed a classifier to differentiate between MDD and control subjects with sensitivity=83% and specificity=93%
2017	Khandoker et al [38]	61^aa^	68^aa^	26-48^aa^	MDD with suicidal ideation	Neuropsychiatric Interview (MINI) version 5 and the Hamilton Depression Rating Scale	N/R	A multivariate logistic regression classifier to differentiate between MDD and control with accuracy=93.3%
2017	Kobayashi et al [40]	55	49	21-59	MDD with paced respiration	N/R	N/R	HR significantly differentiates between MDD and control subjects with *P*<.05
2016	Khandoker et al [37]	61	68	22-40	MDD with suicidal ideation	Neuropsychiatric Interview (MINI) version 5 and the Hamilton Depression Rating Scale	N/R	A decision tree classifier to differentiate between MDD and control with accuracy=93.4%
2014	Akar et al [31]	39	43.5	16-29	Schizophrenia	DSM-IV criteria	Subjects with diabetes mellitus, hypertension, respiratory diseases, cardiovascular diseases, and comorbidity were excluded	HRV significantly differentiates between patients with schizophrenia and control subjects with *P*<.05
2014	Clamor et al [32]	115	41.7	25-42	Psychosis and depression	Structured mini-international neuropsychiatric interview and community assessment of psychic experience	N/R	HRV was found for psychosis, with significant differences to healthy controls (all *P*≤.007) and to depression (all *P*≤.004),
2013	Minakuchi et al [46]	31	100	20-24	Mental stress	Profile of Mood States	N/R	LF/HF ratio significantly associated with Profile of Mood States with *P*<.01
2013	Lee et al [43]	5	N/R	N/R	State anxiety and trait anxiety	STAI	N/R	N/R
2013	Pham et al [52] (Second experiment)	35^ab^	N/R	N/R	Depression	N/R	N/R	Automated detection of mental illness is feasible using k-nearest neighbours with a sensitivity of 99.03%^aa^ and specificity of 95.09%^aa^
2012	Pham et al [51] (First experiment)	308	N/R	N/R	Depression	N/R	N/R	Automated detection of mental illness is feasible using support vector machines with a sensitivity of 99.78% and specificity of 99.28%

^a^F: female.

^b^4DSQ: four-dimensional symptom questionnaire.

^c^TST: total sleep time.

^d^MVPA: moderate-to-vigorous physical activity.

^e^HRV: heart rate variability.

^f^SCID-II: Structured Clinical Interview for Diagnostic and Statistical Manual of Mental Disorders–IV-Axis II.

^g^CGI-S:

^h^GAF: global level of functioning.

^i^HR: heart rate.

^j^PHQ-9: Patient Health Questionnaire–9.

^k^N/R: not reported.

^l^TSST: Trier Social Stress Test.

^m^MDD: major depressive disorder.

^n^SDS: Self-Rating Depression Scale.

^o^HDRS: Hamilton depression rating scale.

^p^BDI: Beck Depression Inventory.

^q^EPDS: Edinburgh Postnatal Depression Scale.

^r^STAI: State-Trait Anxiety Inventory for Adults.

^s^LF: low frequency.

^t^HF: high frequency.

^u^DASS-21: Depression Anxiety Stress Scales–21.

^v^STAI-Y: State-Trait Anxiety Inventory.

^w^SWLS: Satisfaction With Life Scale.

^x^SAD: social anxiety disorder.

^y^DSM-5: fifth edition of the Diagnostic and Statistical Manual of Mental Disorder.

^z^LSAS: Liebowitz Social Anxiety Scale.

^aa^These values correspond to the dataset that the same authors reported in [37]

^ab^The values presented here pertain to the second experiment that is reported in this journal paper. The first experiment, which the same authors had published earlier in a conference paper [51], is different from the one presented here.

### Depression

Several studies have evaluated the use of PPG for diagnosing depression [32-34,37,38,40,41,44,45,48,49,52,54]. A study in Japan evaluated the use of finger PPG for major depressive disorder (MDD) and found that it could accurately detect it, with a very high correlation with electrocardiogram (ECG)-based HRV measures [33]. This included the high frequency (HF) component of HRV measures and the ratio of low frequency (LF) to HF, which is known as LF/HF HRV. The sensitivity and specificity for diagnosing MDD were 83% and 93%, diagnosing MDD [33].

In addition to finger-based PPG methods, face-based (video) methods have also been found to be accurate for diagnosing depression. A study in Japan found that a PPG web camera–based system that detects blood flow to the face (arteries in the sinus region) had a sensitivity of 73% and specificity of 85% for diagnosing MDD [54]. A considerable correlation was also found between depression scores (subjective self-report) and video-based PPG assessments of MDD. This technology has also been correlated with ECG findings [54]. Similarly, another study in Japan also found an 86% sensitivity in assessing MDD during exercise when compared with controls, although it was only 68% when exposed to a mental stress test [40].

The most advanced PPG technology integrates video- and finger-based PPG methods, using smartphones that simultaneously make use of both. A study in China found that smartphone-based PPG could be used to assess happiness, as subjectively reported that life satisfaction was positively correlated with an HRV index, specifically the root mean square of beat intervals (RMSDD) and negatively correlated with log HF and log LF. There was also a negative correlation between these HRV indices and depression scores [44]. Another study also found a considerably negative relationship between autonomic reactivity assessed via PPG and MDD and that this relationship was considerable when compared with controls without MDD [42].

Interestingly, one study evaluated brain changes in depression and compared them with HRV. Researchers found a positive association between RMSDD, cortical thickness, self-reported depression, and HF HR [41]. Furthermore, a study in Germany found that patients who are depressed with psychosis had significantly lower RMSDD and SD of the N-N intervals than controls, whereas their HR and LF/HF ratio were significantly higher than in controls [32]. Similar findings with respect to increased RMSDD, SD of the N-N intervals, and LF/HR ratios were noted in patients who are depressed with MDD in Thailand relative to controls, and cortisol levels were also elevated [49]. Thus, there is a growing body of evidence suggesting that there are differences in HRs among patients with depression relative to healthy controls. In addition, these objective brain imaging data support the changes noted in patients with depression using HRV [41].

### Anxiety Conditions

Most studies on anxiety used validated anxiety measures, such as the State-Trait Anxiety Scale [55], the Generalized Anxiety Disorder [56], and the Depression Anxiety Stress Scale [57,58], to validate their findings alongside PPG assessments. Most studies have involved comparisons between relaxation exercises and subsequent psychological stress-inducing exercises. In general, HR increased during stress-provoking tasks and declined during relaxation across the studies (Celka et al [10], Kontaxis et al[42], Lee et al [43], Chen et al [64], Panganiban et al [65]). Physiological distress responses in HR were similar for both anxiety and stress, with increases in HR across studies. A study in Canada found a strong association between self-reported anxiety and stress and subsequently elevated HR using smartphone-based PPG assessment [35]. Another study evaluated the Oura Ring, which is a commercially available wearable sleep and activity tracker (Oura Health, Oulu, Finland), and found that self-reported anxiety was positively associated with HRV [48]. There was also a high agreement between the Oura Ring PPG-based HRV assessment and ECG (*r*=0.98) [48].

A study in pregnant women found that they had significantly lower levels of LF if they had obsessive-compulsive disorder, whereas LF was elevated in panic disorders, social anxiety, and social phobias [39]. The LF/HF ratio was also elevated in pregnant women with phobias and panic problems [39]. Furthermore, the LF/HF ratio was elevated in participants who experienced stress after taking a stress-inducing test, most often involving arithmetic, with a few studies finding a strong positive relationship between the LF/HF ratio and stress [43,46,50]. One study specifically focused on anxiety and found a positive relationship between the LF/HF ratio and anxiety based on self-assessments using the State-Trait Anxiety Scale [50]. HR [34,35] and RMSDD increase during stress [46].

### Validity

Several studies have found good agreement between ECG-based HRV indices and PPG and anxiety [33,36,48,50]. One study found that PPG had a sensitivity of 83% and a specificity of 93% for assessing anxiety. It was also found that ECG and PPG had perfect agreement (*r*=1.0) [33]. Another study found a high correlation (*r*=0.81) between self-reported anxiety and anxiety assessment using PPG [50]. A study on social anxiety disorder found that HR could predict the condition with 88.9% accuracy [36]. The study which evaluated the Oura Ring for anxiety found a high correlation between EEG and PPG findings (*r*=0.98) [48]. In addition, a study pointed out that PPG signaling is the most accurate when participants are relaxed rather than under stressful conditions [10].

### Other Mental Health Problems and Symptoms

One study evaluated the use of PPG to assess the symptoms of suicidal ideation in patients with depression. By examining diastolic and systolic entropy lag scores, the researchers found that PPG has an accuracy of 96.7% in diagnosing suicidal ideation in patients with MDD by examining diastolic and systolic entropy lag scores [38]. The researchers also found that the scores of patients on the Hamilton Depression Scale, which they completed via self-reports, were highly correlated (*r*=0.96) with the entropy lag scores [38].

Besides suicidal thoughts, a few other studies have evaluated PPG for less prevalent mental health problems, including schizophrenia and borderline personality disorder. One study evaluated the use of PPG in schizophrenic patients and found that patients with schizophrenia had elevated LF/HF ratios compared with controls but reduced HF while resting (*P*<.05). However, HRV was reduced overall in patients during the stress and subsequent relaxation phases, with lower RMSDD and SD of the N-N intervals than in their counterparts without schizophrenia [31]. In addition, one study evaluated borderline personality disorders by using PPG. The researchers found that patients with a higher resting HR also had improved recovery when evaluated longitudinally over a 2-year period [53].

### Study Quality

Most studies were conducted with small sample sizes. Ten out of 24 studies had a small sample size of <50 participants, ranging from 5 [43] to 40 [49] participants. The largest study was carried out in 2012 to assess depression using fingertip PPG–based technology, with a total of 308 participants [51,52]; however, sex (male or female) was not reported. A recent study [45] targeted the same research point, assessing depression using fingertip PPG in 36 participants. The first study on N=308 scored 99.53%, whereas the latter on N=36 achieved 85.3% accuracy.

Only 8 patients had a clinical diagnosis [33,36,42,47,51,52,54]. Only 3 studies compared patients with MDD with those who did not have clinical depression [42,52,54]. One study examined suicidal ideation in patients with MDD and in patients with MDD who did not have suicidal ideation [37]. There were no longitudinal studies. Randomization, allocation concealment, and blinding were not conducted in the interventions with the 2 arms.

## Discussion

### Principal Findings

This review aimed to evaluate the use of PPG in assessing and screening (including prescreening) mental health problems. We found an association between increased HR and mental health outcomes. The mental health literature indicates that depression and anxiety increase the risk of morbidity and mortality from heart disease [16,17], and the studies in this review confirmed changes in HRV in patients with mental health problems. However, none of the studies specifically used PPG-based technologies to screen patients for mental health problems or to monitor symptoms during treatment.

Although the studies were not equal in terms of quality and sample size, a few studies reported good accuracy in detecting mental health conditions; for example, Long et al [45] achieved an accuracy of 85.3% over 36 participants after using the Patient Health Questionnaire–9 as the gold standard for detecting depression. Ismail et al [36] included 34 participants, using social anxiety disorder as a gold standard, for detecting stress. All the developed detectors rely on changes in PPG-based HRV measurements in patients with depression and anxiety. This includes differences between stressful states or feelings of anxiety and relaxation states. However, we did not find any studies that monitored the progress, including symptoms, in patients undergoing treatment. Future studies should consider evaluating the use of PPG to monitor disease progression. However, it remains unclear whether PPG can distinguish between patients on and off medication or changes in mood without medication use.

PPG was also found to be comparable with ECG in assessing the changes in HRV indices associated with mental health outcomes. PPG was positively correlated with self-reported measures of mental health end points overall in this review. HR was elevated when participants were stressed, and PPG could accurately capture these physiological changes, including HR reduction during relaxing exercises [10,42,43,64,65]. In addition to stress, PPG also accurately detects patients who are depressed [32-34,37,38,40,41,44,45,48,49,52,54], including those with MDD. In other words, depression was associated with a lower HR in contrast to stress. This was the case when studies compared patients with MDD with those without MDD, highlighting the ability of PPG to distinguish between clinically depressive and nondepressive states. We were also able to differentiate between patients with clinical depression who had symptoms of suicidal ideation and those who did not.

These findings are relevant given that patients with clinical depression may be monitored for symptoms of suicidal ideation and may quickly receive emergency medical care when evaluated in remote and rural settings. It is also crucial that PPG can rapidly assess anxiety and depression because patients may be quickly referred to medical doctors and receive a timely diagnosis. In addition, access to mental health care is a problem for many people, especially those living in rural and remote areas [11,59]. Virtual PPG mental health screening and monitoring may enable access to medical care for those who do not typically have access to treatment. PPG can be used as a prescreening tool to screen and prioritize medical accessibility in rural patients [20. It is also important for medical practitioners to obtain HR data to obtain more objective mental health measures than subjective self-reports.

We found that the PPG technology has advanced in recent years. Not only are probe-based methods used, but PPG may also be integrated into smartphone technology, which has important implications for accessibility, given the ubiquitous nature of cell phones. However, we identified only a few studies that used smartphones for PPG, highlighting a gap in the research literature and the need for more studies. It appears to be a practical tool, given that patients can easily access technology. Face-based methods using mobile health to detect changes via facial imaging are simple, noninvasive, and practical for users.

General studies on psychological stress in the literature have also found good agreement between PPG- and ECG-based HRV findings, which is applicable to anxiety studies [60-67]. The accuracy in these studies ranged from 77.6% in smartphone-based (CorSense) PPG technology to 98% using a wrist-based method [64,65]. The correlation also varied according to HRV indices when compared with ECG including RMSDD (*r*=0.97) and LF/HF ratio (*r*=0.87) [66]. A PPG wrist-worn technological study found an overall accuracy of 83%, assessing calm states 67% of the time and stressed states 72% of the time [63]. However, one study that examined PPG in the earlobe did not find good agreement with the reference method [61]. Interestingly, a study that used smartphone-based PPG technology and a thermal camera found that the readings were the most accurate when both methods were combined [60]. One study validated their findings by comparing the Huawei PPG watch sensor with not only self-reports but also laboratory measures of cortisol [62]. Another study that used 3 LED lights on the wrist recommended utilizing light green instead of infrared for PPG-based stress detection [64].

### Gold Standard

Currently, there is no widely accepted gold standard for assessing depression or anxiety using PPG. Although PPG has shown promise as a tool for measuring physiological arousal levels and has been used in a number of studies to investigate the physiological changes associated with depression and anxiety, its use as a diagnostic tool is still in its early stages of development. Therefore, a gold-standard tool must be used in combination with PPG.

The studies used self-reported questionnaires and interviews to assess mental health. For example, in terms of interviews, Clamor et al [32] used the Structured Mini-International Neuropsychiatric Interview, Khandoker et al [37] used Neuropsychiatric Interview (MINI) version 5, and Sigrist et al [53] used the Structured Clinical Interview for DSM-IV-Axis II. In terms of self-reported questionnaires, the following instruments were used: Four-Dimensional Symptom Questionnaire, Patient Health Questionnaire–9, Trier Social Stress Test, Self-Rating Depression Scale, Hamilton Depression Rating Scale and Beck Depression Inventory II, Edinburgh Postnatal Depression Scale and the State-Trait Anxiety Inventory for Adults, State-Trait Anxiety Inventory, Depression Anxiety Stress Scales, Satisfaction With Life Scale, DSM-5 and Liebowitz Social Anxiety Scale, Wisconsin Card Sorting Test, Clinicians’ Rating of Depression Severity and Beck Depression Inventory II, and Profile of Mood States. These self-report measures can provide valuable information about an individual’s subjective experiences of depression or anxiety. A few studies [32,37,53] have used a combination of self-report measures and self-reported questionnaires, along with PPG. Five studies did not use the gold standard method, interviews, or questionnaires [33,40,47,51,52].

### Limitations and Ethical Considerations

Despite the outcomes of this review, the results should be interpreted with caution. PPG is an emerging technology that cannot be used as a stand-alone method to assess or monitor mental health. It may also be used as an adjunct along with skilled mental health practitioners. Important factors to consider when diagnosing mental health problems include the severity of symptoms [68] and functional impairment [69], which cannot be detected presently according to the reviewed studies. Furthermore, many studies did not use a comparison group, which is another limitation of this study.

Additionally, it may be difficult to determine whether someone with a mental health problem experiences general life stress or whether their anxiety and stress are directly a result of their mental health condition given the variety in stressful exposures across the studies and similarity in results. Moreover, many studies involved a stressful task that included changes in physiology (getting up after sitting), mental tasks, or stress invoking exposures [42,43,46,47,50,60,64,65]. The studies did not discern between anxiety and anxious states in general. The key challenge is to discern between patients experiencing clinical anxiety, general personal stress, and feelings of anxiety or anxiety common and everyone, not only in clinical populations. Thus, it may also seem that the technology may be most useful in assessing mood changes in patients with depression, as the altered HRV in depression seen in the reviewed studies may be differentiated from stressed or anxious states.

Ethical issues must be considered when implementing PPG technology for mental health applications. Health ethics involves a consideration of harm and benefits [70]. Maximizing the benefits and potential harm is a prima facie ethical duty [70]. This could include the consideration of PPG-associated false positives or negatives when screening for suicidal symptoms or assessing changes in mood states in patients with depression. Thus, clinicians should be mindful of the limitations of this emerging technology and use it prudently along with their clinical practice, backing up findings when possible, through clinical assessment.

### Design Bias

More studies with larger sample sizes and in clinical populations are needed before PPG can be used as an adjunct in assessing mental and psychological health. We found several limitations in the studies we analyzed, including the small number of studies in clinical populations and small sample sizes. There is also a need for randomized controlled trials that may follow up patients. There is a gap in the literature, and randomized controlled trials are needed to validate PPG technology. Patients should also be blinded in future studies to reduce the potential for placebo effect and bias when comparing subjective self-report measures with PPG. They could be blinded by providing them with a similar-looking application on their smartphones and by requiring them to look at the camera and place their fingers on their phones.

### Ethnic Bias

It is worth noting that a recent study [71] revealed that PPG produces systematically higher saturation values in Black patients than in White patients. Participants with darker skin pigmentation may be at an increased risk of unrecognized health conditions. Therefore, collecting and testing PPG signals from Black participants is required to reduce bias [72]. None of the studies recognized or tested this bias in the assessment of mental health. Therefore, we strongly recommend creating publicly available databases with more balanced ethnicities (ie, participants with different pigmentation colors).

### Confounding Bias

Confounds such as hospitalization or the use of pharmacological treatments are essential to control the study design and analysis. None of the included studies reported hospitalization or pharmacological treatments; however, 5 out of 24 studies [31,34,39,41,53] paid attention to possible confounding factors.

In fact, it is essential that all the studies used HR and HRV indices, which can be affected by a wide range of physical and psychological factors [73]. It is also crucial for PPG-based mental health studies to ensure that participants remain in a stable state before data collection. This may involve restricting data collection to a specific time of the day or requiring participants to avoid stressful or physically demanding activities before data collection [74].

Another factor to consider is that other conditions or exposures, such as COVID-19, may affect HRV [75,76]. Thus, clinicians should be mindful of this possibility and rule out COVID-19 or associated comorbidities when using PPG for mental health assessments.

By carefully controlling for these confounding factors and selecting study participants, PPG-based mental health studies can provide less bias about the physiological changes associated with depression and anxiety, helping to advance our understanding of these conditions and the potential of PPG as a diagnostic tool.

### PPG-Based Technology Versus Traditional Mental-Health Assessment Tools

None of the articles included in this study discussed the use of PPG versus traditional mental health assessment tools. PPG is generally considered less invasive than answering questions via interviews or questionnaires, depending on the individual user’s perspective.

We speculate that answering questions (via interview or filling out a questionnaire) about their mental health may make them feel more personal and intimate than having their physiological arousal levels measured through PPG. They may feel more comfortable disclosing their thoughts and feelings through self-report measures, rather than monitoring their physiological responses.

PPG has the potential to offer new insights into the mechanisms underlying mental health disorders. PPG also has a considerable advantage as it provides real-time data acquisition, enabling mental health practitioners to monitor changes in physiological arousal levels. This can be particularly useful in situations where quick and effective interventions are needed. One major concern with this advantage is privacy; users may not feel comfortable when their physiological signals are monitored regularly [77]. They may worry about who has access to the data collected through PPG and how the data will be used.

At this stage, it is debatable what would be more effective and satisfy user preferences. The authors feel that this area is worth investigating to improve mental health assessments in the near future.

### Conclusions

PPG for mental health assessment is an emerging technology with great potential. Currently, it cannot be used to diagnose mental health and psychological problems; however, future trials may provide sufficient evidence for the use of this technology as an adjunct or complementary tool for mental health assessment alongside conventional methods. With the increasing prevalence of telemedicine and remote patient monitoring, PPG has the potential to provide mental health practitioners with cost-effective and convenient means of monitoring their patients’ physiological arousal levels. This can enable earlier detection of mental health issues and enable timely and effective interventions.

## Abbreviations

DSM-5: fifth edition of the Diagnostic and Statistical Manual of Mental Disorders
ECG: electrocardiogram
HF: high frequency
HR: heart rate
HRV: heart rate variability
LF: low frequency
MeSH: Medical Subject Headings
MDD: major depressive disorder
PPG: photoplethysmography
PRISMA-P: Preferred Reporting Items for Systematic Review and Meta-Analysis Protocols
RMSDD: root mean square of beat intervals
